# Healing of a fracture with a significant bone gap in a supradiacondylar fracture of the distal femur treated with dual plating assisted by the use of Nanostructured Hydroxyapatite (NanoBone®) and Hydroxyapatite and Tricalcium Phosphate Blocks (TRIHA+®): Case report

**DOI:** 10.1016/j.tcr.2025.101273

**Published:** 2025-11-15

**Authors:** Francesco Maria Milella, Giovanni Longo, Massimiliano Paolocci, Emanuele Persi, Riccardo Mezzoprete

**Affiliations:** a Azienda Ospedaliera San Camillo de Lellis – Orthopaedics and Traumatology department, Viale J.F. Kennedy, 02100, Rieti, Italy; b Orthopaedics and Traumatology Division - Department of Anatomical, Histological, Forensic Medicine and Orthopaedics Sciences, University “La Sapienza”, Piazzale Aldo Moro 5, 00185, Rome, Italy

**Keywords:** Distal femur fracture, Dual plating, Bone graft substitute, NanoBone®, TRIHA+®

## Abstract

Distal femur fractures present a surgical challenge due to high rates of varus collapse, nonunion, and delayed healing. In cases of severe bone loss, achieving stable fixation while promoting osteogenesis remains critical. The combination of dual plating with advanced bone substitutes may improve outcomes in complex supracondylar fractures.

## Introduction

Distal femur fractures remain a surgical challenge, with a high rate of varus collapse (26.7 %) [1], nonunion, and delayed healing (5–20 %) [2]. The most common risk factors for nonunion include metaphyseal comminution, fractures treated with ORIF, and open fractures [3]. Metaphyseal comminution is an independent risk factor for nonunion in distal femur fractures [4]. Higher nonunion rates have been demonstrated in Arbeitsgemeinschaft für Osteosynthesefragen/Orthopaedic Trauma Association (OTA/AO) fracture types 33.A3, C2, and C3 [5]. Distal femur fractures are rare injuries, with an estimated annual incidence of 8.7/100,000 per year [6], predominantly affecting the geriatric and osteoporotic population [6,7], with a one-year mortality rate ranging from 13.4 % to 22 % depending on the study [6,8]. The use of dual medial and lateral plating for these fractures, although a relatively recent concept, has been widely implemented with promising results [9,10]. Dual plating has shown reduced risk of malalignment in diaphyseal fractures, greater stability in distal and periprosthetic fractures, high bone healing rates in cases of nonunion, and relatively low complication rates compared to other techniques [11].

As an adjunct in the healing process of multifragmentary fractures, there is growing interest in the use of osteoinductive materials composed of nanostructured hydroxyapatite (HA), incorporated into a silica gel matrix and suspended in a hydrogel/polymer silica carrier (NanoBone® Bone Graft Substitute, Artoss GmbH, Rostock, Germany) [12], as well as osteoconductive materials such as hydroxyapatite and tricalcium phosphate blocks (TRIHA+®) [13].

This study presents the case of a patient with a right distal femur supradiacondylar fracture, AO type 33 C3, with a severe bone gap, successfully treated using dual plating combined with a mixture of NanoBone® and TRIHA+®.

## Case report

**Fig. 1:**
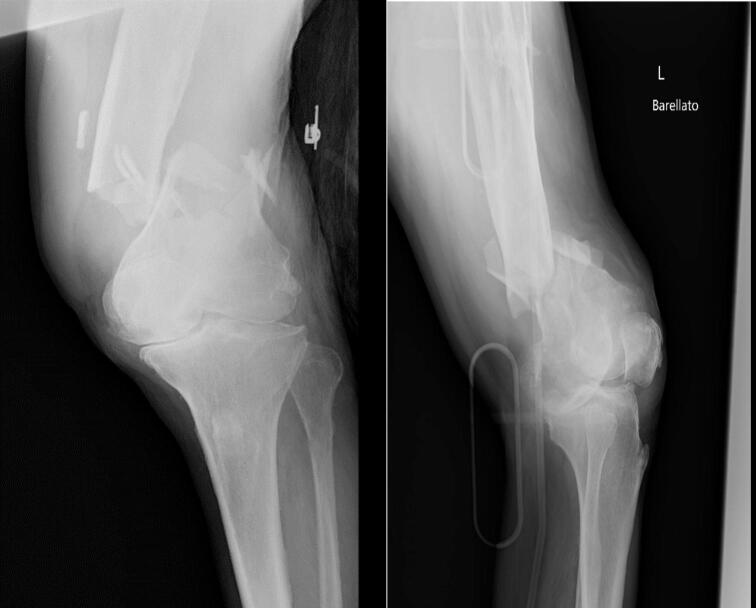
X-rays at the entrance to the emergency room.

**Fig. 2:**
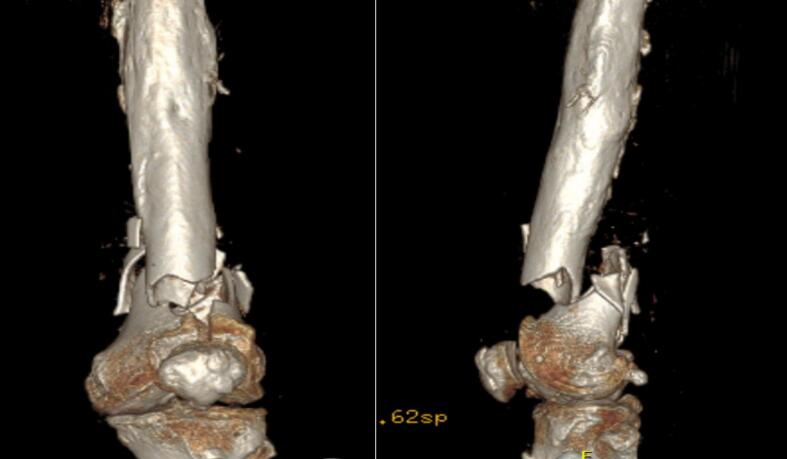
CT at the entrance to the emergency room.

A 78-year-old male patient presented to our emergency department in the early hours of April 27, 2024, following a domestic fall. Trauma assessment revealed a left supradiacondylar femur fracture, AO type 33 C3 (Fig. 1, Fig. 2). The patient's medical history included type II diabetes mellitus, severe heart disease with pacemaker implantation, anticoagulation therapy with apixaban, and a previous ipsilateral distal femur metaphyseal fracture treated 50 years earlier with a plate and screw, removed approximately two years after implantation.

On physical examination, the patient had a swollen, painful left knee with tenderness at the fracture site. Despite intact skin, a significant femoral axis deformity was noted. No peripheral vascular or neurological deficits were recorded. Skeletal traction via a trans-tibial pin was applied by the on-call orthopedic surgeon, and the patient was admitted for surgical treatment.

On May 2, 2024, after obtaining informed consent and conducting anesthetic and cardiology evaluations, the patient underwent surgical reduction and internal fixation. As per our institutional protocol, preoperative antibiotic prophylaxis was administered with 2 g of intravenous cefazolin 30 min before incision. No tourniquet was used.

The selected surgical approach was a modified anterolateral knee Swashbuckler approach [14], with the patient in a supine position and the knee flexed at 30° to neutralize muscle forces. This approach allowed medial patellar displacement for improved joint visualization. Once the joint plane was accessed, the fracture lines were reduced using a reduction clamp and stabilized with two cannulated screws. After achieving absolute stability with anatomical reduction of the articular portion, the metaphyseal segment was aligned, restoring axis, rotation, and length. Provisional fixation was performed using a lateral bridge plate (Technovare).

**Fig. 3:**
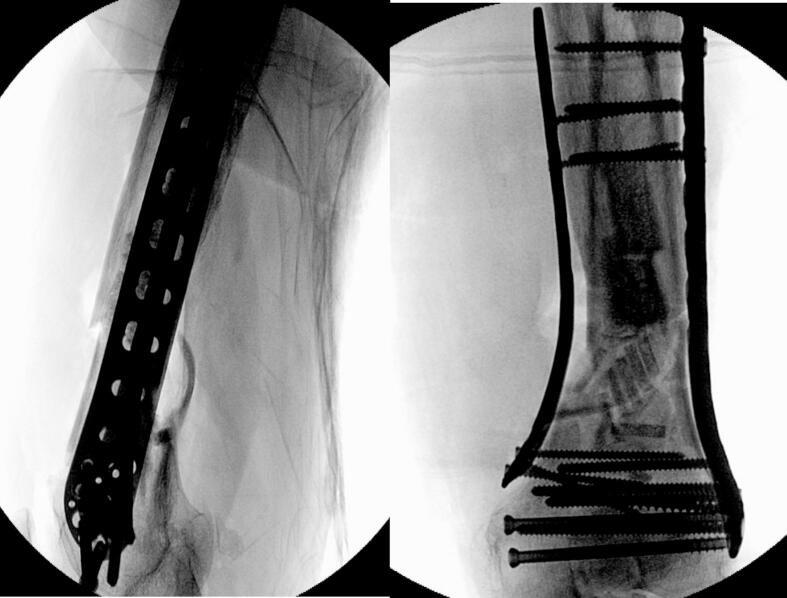
Intraoperative shots.

**Fig. 4:**
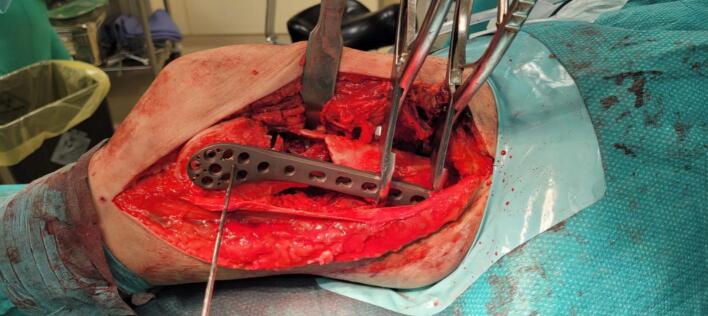
View of the bone gap.

**Fig. 5:**
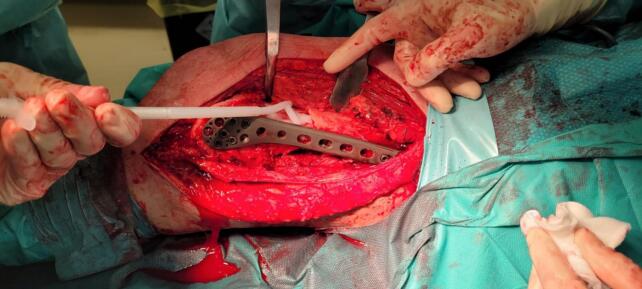
Using nanobone as a filler putty.

**Fig. 6:**
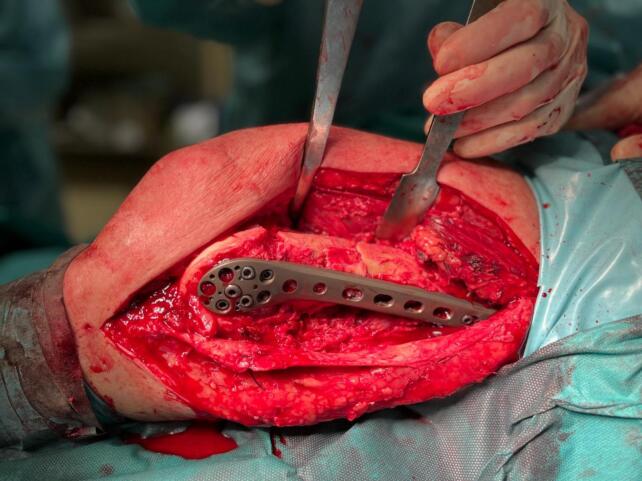
Final effect of bone gap closure.

Due to severe comminution and significant bone loss, additional stability was required to prevent varus collapse. Thus, a tibial plate (Technovare) was used as a medial distal femur plate via a minimally invasive percutaneous osteosynthesis (MIPO) approach. Given the substantial anterior-lateral cortical bone loss, the bone gap was filled with TRIHA+® hydroxyapatite and tricalcium phosphate blocks, bonded together using NanoBone® silica gel. The silica gel was also used to fill residual voids (Fig. 3, Fig. 4, Fig. 5, Fig. 6).

The patient received low-molecular-weight heparin anticoagulation therapy 12 h postoperatively, continuing until full weight-bearing was resumed. No surgical drains were placed. Sutures were removed two weeks after surgery. No postoperative complications occurred.

The rehabilitation protocol included active and passive knee flexion-extension starting on postoperative day one, with progressive quadriceps strengthening. Full weight-bearing was restricted for three weeks, followed by partial weight-bearing at 20 kg using two crutches for an additional three weeks, then progressive weight-bearing as tolerated from week six onward.

**Fig. 7:**
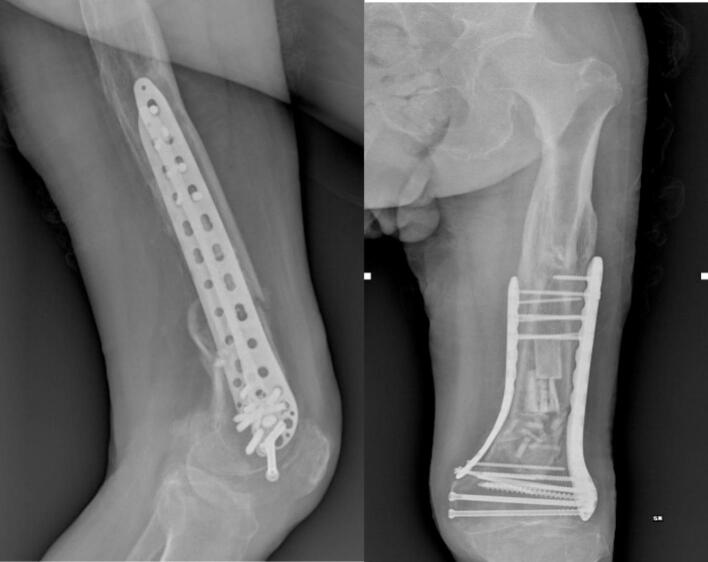
Control X-ray six months after surgery.

**Fig. 8:**
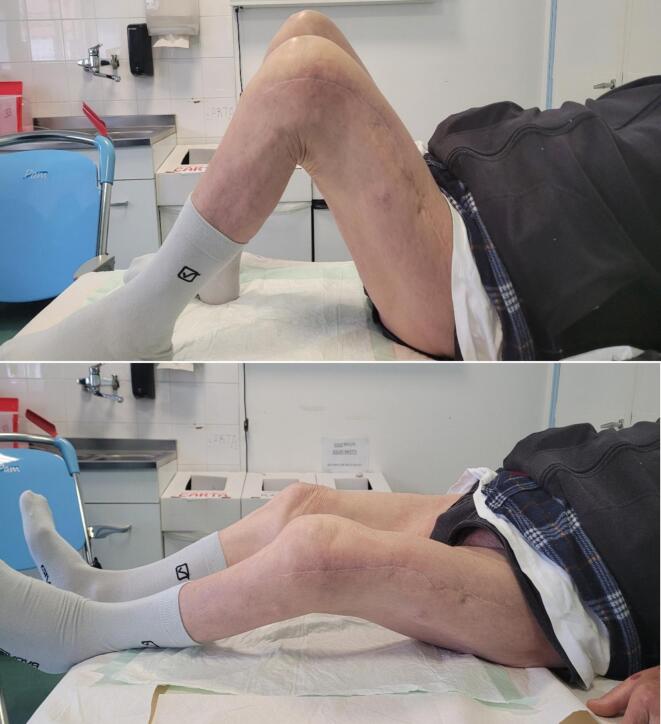
Clinical control six months after surgery.

At the six-month follow-up, clinical evaluation demonstrated a well-healed surgical incision, absence of varus collapse, and satisfactory callus formation. The patient ambulated without a limp, and knee range of motion was comparable to the contralateral side, with no evidence of joint crepitus (Fig. 7, Fig. 8). At this stage, the patient's KOOS sub-scores were as follows: Pain = 92, Symptoms = 88, ADL = 94, Sport/Rec = 70, and QoL = 82.

**Fig. 9:**
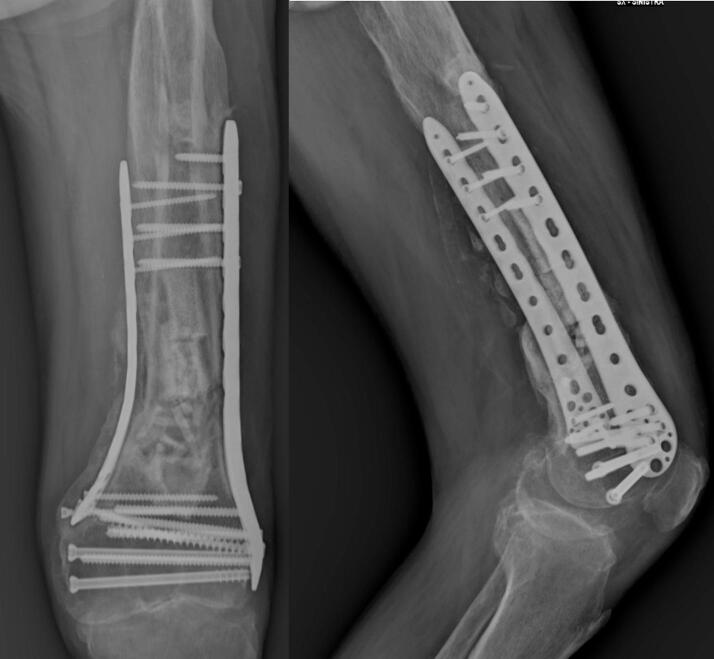
Follow-up X-ray approximately one year after surgery.

At approximately one year postoperatively, radiographic assessment confirmed complete fracture consolidation, a well-healed surgical site, and no signs of varus collapse, with ongoing callus remodeling (Fig. 9). On clinical examination, the patient remained free of a limp, and knee range of motion was comparable to the contralateral limb, although a mild restriction was also observed in the opposite knee.

At this final follow-up, the patient's KOOS sub-scores were: Pain = 94, Symptoms = 90, ADL = 96, Sport/Rec = 72, and QoL = 85. These findings indicate an improvement compared with the six-month assessment, reflecting further pain reduction, fewer residual symptoms, and excellent recovery of daily function. The lower Sport/Rec score is consistent with age-related limitations, while the QoL domain reflects a favorable perception of knee-related quality of life.

## Discussion

We report the case of a distal femur supracondylar fracture, AO type 33 C3, treated with dual medial and lateral plating, assisted by hydroxyapatite and tricalcium phosphate blocks (TRIHA+®) and nanostructured hydroxyapatite incorporated in a silica gel matrix (NanoBone®) as a bone void filler.

We believe that both the dual plating strategy and the use of bone substitutes played a crucial role in achieving stable fixation and promoting biological healing. While the osteoconductive properties of hydroxyapatite and tricalcium phosphate are well established, most evidence on the osteoinductive potential of NanoBone® derives from preclinical studies and dental applications [15]. In our experience, the combination of these materials created an optimal environment for bone regeneration, supporting callus formation and enhancing integration at the fracture site.

The favorable clinical and functional outcomes observed — including good range of motion, absence of mechanical complications, and progressive improvement in patient-reported knee function — likely reflect both the mechanical stability provided by dual plating and the biological support offered by the bone substitutes. Furthermore, the patient's early mobilization and adherence to rehabilitation contributed to preserving joint mobility and minimizing functional deficits.

These findings suggest that the integration of novel biomaterials into standard osteosynthesis techniques can improve outcomes in complex distal femur fractures, even in elderly patients. Further clinical research is warranted to validate these observations and to better define the indications and long-term benefits of such biomaterials in orthopedic trauma surgery.

## CRediT authorship contribution statement

**Francesco Maria Milella:** Writing – review & editing, Writing – original draft, Visualization, Validation, Supervision, Software, Resources, Project administration, Methodology, Investigation, Funding acquisition, Formal analysis, Data curation, Conceptualization. **Giovanni Longo:** Writing – review & editing, Validation, Resources, Methodology, Investigation, Funding acquisition, Formal analysis, Data curation. **Massimiliano Paolocci:** Supervision. **Emanuele Persi:** Supervision, Methodology. **Riccardo Mezzoprete:** Supervision.

## Informed consent

All the patients gave their approval via informed consent to publish their clinical and laboratory data, within utterly lawful respect of privacy.

## Ethical approval

Not required.

## Funding

No grant has been received for this study.

## Declaration of competing interest

The authors have no financial interest to declare in relation to the content of this article.
